# Supplementary material for: The role of sex and femininity in preferences for unfamiliar infants among Chinese adults
Source: PLoS One. 2020 Nov 12;15(11):e0242203. doi: 10.1371/journal.pone.0242203 (PMC7660579; doi:10.1371/journal.pone.0242203)
Supplement: S1 Table — (DOCX) [file pone.0242203.s001.docx]

**S1 Table. Sample size broken down by sex, marital status and ethnicity**

| Sex | Marital status | | Ethnicity | | | |
| --- | --- | --- | --- | --- | --- | --- |
|  | Married | Unmarried | Han | Miao | Dong | Others |
| Female | 100 | 54 | 77 | 37 | 16 | 24 |
| Male | 97 | 49 | 77 | 38 | 16 | 15 |
| Total | 197 | 103 | 154 | 75 | 32 | 39 |

Note. Others included Tujia, Buyi, Bai, Chuanqing, Gejia, Gelao, Li, Man, Hui, and Mulao. Each of these minority groups included less than 10 people.
