# Supplementary material for: The role of sex and femininity in preferences for unfamiliar infants among Chinese adults
Source: PLoS One. 2020 Nov 12;15(11):e0242203. doi: 10.1371/journal.pone.0242203 (PMC7660579; doi:10.1371/journal.pone.0242203)
Supplement: S2 Table — (DOCX) [file pone.0242203.s002.docx]

**S2 Table. Details of ANOVA**

| Dependent variables | Sex | | Marital status | | Ethnicity | | Interactions | |
| --- | --- | --- | --- | --- | --- | --- | --- | --- |
|  | *F* | *η^2^* | *F* | *η^2^* | *F* | *η^2^* | *F* | *η^2^* |
| Femininity | 1.545 | 0.012 | 0.253 | 0.001 | 1.995 | 0.021 | 0.137-1.545 | 0.000-0.016 |
| Masculinity | 36.818^***^ | 0.115 | 2.733 | 0.010 | 0.575 | 0.006 | 0.001-1.117 | 0.000-0.012 |
| Interest in infants | 9.716^**^ | 0.033 | 2.782 | 0.010 | 1.405 | 0.015 | 0.001-1.249 | 0.000-0.013 |
| Liking | 0.593 | 0.002 | 2.236 | 0.008 | 1.848 | 0.019 | 0.396-2.017 | 0.002-0.021 |
| Representational | 3.479 | 0.012 | 2.121 | 0.007 | 0.082 | 0.001 | 0.252-1.942 | 0.003-0.013 |
| Evoked | 1.100 | 0.004 | 1.075 | 0.004 | 0.289 | 0.003 | 0.152-1.032 | 0.001-0.011 |

Note. ^**^ *p*<.01, ^***^ *p*<.001. The following table is the same.
