# Supplementary material for: The role of sex and femininity in preferences for unfamiliar infants among Chinese adults
Source: PLoS One. 2020 Nov 12;15(11):e0242203. doi: 10.1371/journal.pone.0242203 (PMC7660579; doi:10.1371/journal.pone.0242203)
Supplement: S3 Table — (DOCX) [file pone.0242203.s003.docx]

**S3 Table. Full summary of 2-level multilevel models of motivation towards infant faces**

| Fixed effects | Liking | | Representational | | Evoked | |
| --- | --- | --- | --- | --- | --- | --- |
|  | Estimate | *t* | Estimate | *t* | Estimate | *t* |
| The emotion-level |  |  |  |  |  |  |
| Neutral | 6.342 | 70.136^***^ | 51.123 | 13.942^***^ | 169.610 | 19.682^***^ |
| Laughing | 1.418 | 20.945^***^ | 40.637 | 13.823^***^ | 106.540 | 14.684^***^ |
| Crying | -1.883 | -22.151^***^ | -70.210 | -17.931^***^ | -147.920 | -18.846^***^ |
| The participant-level |  |  |  |  |  |  |
| Sex | 0.327 | 1.505 | 10.173 | 1.179 | 29.120 | 1.476 |
| Age | 0.012 | 0.470 | 1.009 | 0.940 | 0.139 | 0.047 |
| MS | 0.410 | 2.239^*^ | 11.077 | 1.413 | 13.802 | 0.758 |
| Ethnicity | 0.441 | 2.488^*^ | 1.845 | 0.246 | 30.357 | 1.789 |
| Fem | 0.102 | 0.464 | 1.321 | 0.173 | 1.277 | 0.065 |
| Mas | 0.108 | 0.589 | -2.073 | -0.317 | 0.223 | 0.017 |
| Mas × Sex | 0.007 | 0.021 | -1.639 | -0.129 | 6.099 | 0.268 |
| Fem × Sex | 0.721 | 2.379^*^ | 30.275 | 2.312^*^ | 83.819 | 2.891^***^ |
| Laughing× Sex | -0.460 | -2.853^**^ | -2.574 | -0.380 | -27.661 | -1.680 |
| Laughing × Age | 0.012 | 0.550 | -0.105 | -0.116 | 1.866 | 0.791 |
| Laughing × MS | -0.092 | -0.655 | -2.005 | -0.313 | -12.596 | -0.800 |
| Laughing × Ethnicity | -0.299 | -2.176^*^ | -13.117 | -2.200^*^ | -53.562 | -3.880^***^ |
| Laughing × Fem | 0.099 | 0.661 | -0.968 | -0.194 | -1.062 | -0.067 |
| Laughing × Mas | 0.149 | 1.315 | 5.468 | 1.255 | 12.709 | 1.037 |
| Laughing × Mas × Sex | -0.044 | -0.215 | 1.930 | 0.220 | 15.052 | 0.747 |
| Laughing × Fem × Sex | -0.151 | -0.647 | -9.450 | -0.906 | -42.868 | -1.810 |
| Crying × Sex | 0.150 | 0.823 | -6.787 | -0.764 | -5.878 | -0.301 |
| Crying × Age | -0.060 | -2.200^*^ | -0.880 | -0.694 | -2.639 | -0.939 |
| Crying × MS | 0.345 | 2.007^*^ | 8.687 | 1.061 | 7.784 | 0.461 |
| Crying × Ethnicity | 0.058 | 0.339 | 11.283 | 1.413 | -10.724 | -0.666 |
| Crying × Fem | -0.234 | -1.185 | -8.046 | -0.754 | -3.280 | -0.172 |
| Crying × Mas | 0.037 | 0.238 | 9.751 | 1.240 | 13.394 | 0.975 |
| Crying × Mas × Sex | -0.406 | -1.642 | -10.412 | -0.856 | -31.190 | -1.211 |
| Crying × Fem × Sex | 0.086 | 0.277 | -3.214 | -0.208 | -22.136 | -0.728 |
